# The predictive power of serum vitamin D for poor outcomes in COVID‐19 patients

**DOI:** 10.1002/fsn3.2591

**Published:** 2021-09-19

**Authors:** Hoda Derakhshanian, Hadith Rastad, Sanjoy Ghosh, Marjan Zeinali, Mahsa Ziaee, Tara Khoeini, Mohsen Farrokhpour, Mostafa Qorbani, Mona Ramezani Ghamsari, Hossein Hasani, Zahra Mirzaasgari

**Affiliations:** ^1^ Department of Biochemistry Genetics and Nutrition School of Medicine Alborz University of Medical Sciences Karaj Iran; ^2^ Dietary Supplements and Probiotic Research Center Alborz University of Medical Sciences Karaj Iran; ^3^ Non‐communicable Diseases Research Center Alborz University of Medical Sciences Karaj Iran; ^4^ Department of Biology Okanagan Campus University of British Columbia Kelowna BC Canada; ^5^ Department of Neurology Firoozgar Hospital Iran University of Medical Sciences Tehran Iran; ^6^ Department of Internal Medicine Firoozgar Hospital Iran University of Medical Sciences Tehran Iran; ^7^ Clinical Research Development Unit Shahid Rajaei Educational and Medical Center Alborz University of Medical Sciences Karaj Iran; ^8^ Department of Community Nutrition School of Nutritional Sciences and Dietetics Tehran University of Medical Sciences Tehran Iran; ^9^ Shefa Neuroscience Research Center Tehran Iran

**Keywords:** coronavirus infections, COVID‐19, SARS‐CoV‐2, vitamin D

## Abstract

Considering the high prevalence of vitamin D deficiency worldwide and its relationship with immune response to viral infections, this study attempted to identify the predictive power of serum vitamin D for poor outcomes among the COVID‐19 patients. This retrospective cohort study included all patients with confirmed COVID‐19 hospitalized between February 20, 2020, and April 20, 2020, at a designated COVID‐19 hospital, located in Tehran province, Iran. General characteristics, medical history and clinical symptoms were recorded by trained physicians. Blood parameters including complete blood count, creatinine, lactate dehydrogenase, creatine phosphokinase, erythrocyte sedimentation rate, C‐reactive protein and vitamin D were tested. This study included 290 hospitalized patients with COVID‐19 (the mean age [*SD*]: 61.6 [16.9], 56.6% males), of whom 142 had vitamin D concentrations less than 20 ng/ml, defined as vitamin D deficiency. COVID‐19 patients with vitamin D deficiency were more likely to die (Crude OR [95% CI]: 2.30 [1.25–4.26]), require ICU (2.06 [1.22–3.46]) and invasive mechanical ventilation (2.03 [1.04–3.93]) based on univariate logistic regression results. Although, after adjusting for potentials confounders such as gender and age, the association between vitamin D and need to invasive mechanical ventilation lost its significance, adjusted values for the risk of death and ICU requirement were still statistically significant. Vitamin D deficiency can be considered as a predictor of poor outcomes and mortality in COVID‐19 patients. Therefore, checking serum 25 (OH) D on admission and taking vitamin D supplements according to the prophylactic or treatment protocols is recommended for all COVID‐19 patients.

## Graphical abstract

According to our study, COVID‐19 patients with vitamin D deficiency were about twice as much at risk for ICU hospitalization and death, even after adjusting the statistical model for age and gender.

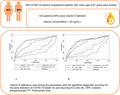

## 1 INTRODUCTION

Since the world has faced the pandemic of Coronavirus disease 2019 (COVID‐19), several studies have been established to elucidate the pathophysiology and treatment of this disease. The epidemiological surveys show that the disease is highly contagious. However, its severity widely ranges from no symptoms to death (COVID C & Team R, 2020; Wu et al., 2020). So, in this situation, understanding the risk factors affecting the patient's response to the novel coronavirus SARS‐CoV‐2 is of great importance. Beside age, gender, underlying illnesses and general nutritional status, vitamin D has been under the spotlight in scientific debates (Grant et al., 2020). Vitamin D is considered a steroid hormone due to its endogenous secretion in the body as well as its ability to regulate genes expression by interacting with its intracellular receptor. This fat‐soluble secosteroid can be provided by few dietary sources such as fish oil, eggs and fortified food. However, it is mostly synthesized in the skin by action of sun ultraviolet radiation 7‐dehydrocholesterol followed by two steps of hydroxylation in liver and kidney, respectively. Calcitriol (1,25‐dihydroxycholecalciferol), the active form of vitamin D, can bind to its nuclear receptor (VDR) and contribute to modifying the cells transcriptional output (Hewison, 2012). Several reviews suggested that, vitamin D plays a role in reducing the risk of viral infections by affecting cellular natural and adaptive immunity (Beard et al., 2011). The antiviral properties of this vitamin are also due to its ability in suppressing the expression of pro‐inflammatory factors, increasing the expression of anti‐inflammatory cytokines, promoting the induction of the T regulatory cells and upregulation of the antimicrobial/antiviral peptides such as cathelicidin and defensins (Greiller & Martineau, 2015; Wei & Christakos, 2015). In addition, vitamin D interacts with the renin–angiotensin system (RAS), which is known to be involved in COVID‐19 infection (Alifano et al., 2020). Considering the high prevalence of vitamin D deficiency worldwide, the present study attempted to identify the predictive power of serum vitamin D for poor outcomes among the COVID‐19 patients.

## 2 MATERIALS AND METHODS

### 2.1 Study design and participants

Our retrospective cohort study included all patients with confirmed COVID‐19 hospitalized between February 20, 2020, and April 20, 2020, at a designated COVID‐19 hospital, located in the province of Tehran, Iran. We excluded patients from the study if they were still hospitalized or their vitamin D level was not measured. The study was ethically approved by the ethics committee of Tehran University of Medical Sciences, Tehran, Iran (Code: IR.ABZUMS.REC.1399.124).

### 2.2 Data collection

Two trained physicians extracted data on the following variables from the medical records of discharged patients: age, gender, medical history, clinical signs, symptoms, O_2_ saturation, requiring invasive mechanical ventilation on admission, the first laboratory findings in the hospital, discharged status (dead or alive), also real‐time polymerase chain reaction (RT‐PCR) test and chest computed tomography (CT) scan findings.

This research adhered to the Declaration of Helsinki guidelines. Research and Ethics Committees of Alborz University of Medical Sciences (ABZUMS) and Iran University of Medical Sciences (IUMS) reviewed and approved the study protocol and waived the requirements for informed consent. To protect confidentiality and anonymity of the patients, a unique identification number was assigned to each included patient.

### 2.3 COVID‐19 diagnosis

COVID‐19 diagnosis was confirmed by one of the following criteria: 1) a positive RT–PCR result or 2) a positive pulmonary abnormality on chest CT based on the radiological criteria of COVID‐19 infection (Ai et al., 2020; Mahdavi et al., 2020).

### 2.4 Laboratory testing

In two aforementioned hospitals, the oropharyngeal swab specimens of patients were collected on admission and examined in predetermined laboratories to detect SARS‐CoV‐2 viral nucleic acid using RT‐PCR assay. Blood parameters listed below were extracted from the first blood tests results for each patient: complete blood count (CBC) including white blood cells (WBC) count and type, hemoglobin (Hb), creatinine, lactate dehydrogenase (LDH), creatine phosphokinase (CPK), C‐reactive protein (CRP), erythrocyte sedimentation rate (ESR), aspartate and alanine transaminases (AST, ALT), prothrombin time (PT), partial thromboplastin time (PTT) and vitamin D.

### 2.5 Comorbidity

On admission, patients were asked whether they had a medical history of the comorbidities including: diabetes mellitus (DM), hypertension (HTN), cardiovascular disease (CVD), cancer, chronic renal failure (CRF; dialysis or non‐dialysis), chronic liver diseases, psychological disorder, chronic pulmonary disease, asthma, thyroid dysfunction, immunodeficiency, autoimmune disease, hematologic disease and neurological disorder.

### 2.6 COVID‐19 Outcomes

Based on the discharge status, patients were classified as cured (survivors) or dead (non‐survivors). Need for intensive care unit (ICU) and need for invasive mechanical ventilation were also considered as criteria for severity of disease. In our hospitals, COVID‐19 patients who met all following criteria will be discharged: lack of fever for at least 72 h, alleviation of respiratory symptoms and improvement in pulmonary abnormalities on chest CT scan.

### 2.7 Statistical analysis

We summarized the general and clinical characteristics of the cohort using appropriate descriptive statistics, mean (standard deviation [*SD*]) or median (interquartile range [IQR]), for continuous variables and frequency (%) for categorical variables. Vitamin D deficiency was defined as the vitamin D concentrations less than 20 ng/ml. Then patients fall into two groups with or without vitamin D deficiency. We compared characteristics of patients with and without vitamin D deficiency using two‐tailed *t*‐tests, Mann–Whitney *U* tests or Chi‐square tests.

The receiver operating curve (ROC) analysis was performed to compare the predictive abilities of vitamin D concentration and other blood parameters for predicting the poor outcomes of COVID‐19; area under the curve (AUC) and its 95% confidence interval (CIs) are reported for each assessed parameter. Univariable and multivariable logistic regression models were used to assess the association of vitamin D deficiency with each poor outcomes of COVID‐19 including death, need to ICU care and receiving invasive mechanical intubation. Of factors associated with vitamin D deficiency, those with a *p‐*value <.2 on univariate analysis were entered into multivariable logistic as a potential confounder. Results are presented as crude and adjusted odds ratio (OR) and 95% CI. We also performed a log‐rank test to determine if there were differences in the survival distribution between males and females and two age groups ≥65 and <65 years. A *p*‐value of less than .05 was considered as statistically significant. All statistical analyses were performed using STATA version11 (Stata Corp LP).

## 3 RESULTS

For the present analyses, 290 COVID‐19 patients who were hospitalized during the study period were included, of whom 142 had vitamin D deficiency. Table 1 presents general characteristics and disease‐related symptoms in the study population on admission, overall and by vitamin D deficiency status. The mean age (*SD*) of patients was 61.6 (16.9) and 56.6% of them (*n* = 164) were male.

**TABLE 1:** General characteristics and disease‐related symptoms in the study population, overall and by vitamin D status

Variables	Total *N* = 290	Without vitamin D deficiency *N* = 148	With vitamin D deficiency *N* = 142	*p*‐value
Demographic				
Age (year), mean (*SD*)	61.6 (16.9)	60.3 (16.1)	63.0 (17.6)	.183
Age ≥65 year, % (*N*)	42.4 (123)	39.2 (58)	45.8 (65)	.257
Gender male, % (*N*)	56.6 (164)	50.0 (74)	63.4 (90)	.022
COVID−19‐related symptoms, % (*N*)				
Cough	48.4 (140)	53.4 (79)	42.9 (61)	.076
Fever	40.3 (112)	39.6 (57)	41.0(55)	.804
Tiredness	41.0 (103)	38.8 (52)	43.6 (51)	.442
P0_2_<93%	55.0 (153)	50.0 (72)	60.4 (81)	.080
Comorbidities, % (*N*)				
DM	31.5 (87)	30.3 (44)	32.8 (43)	.658
HTN	34.2 (95)	36.3 (53)	31.8 (42)	.431
CVD	21.6 (59)	21.4 (31)	21.9 (28)	.921
Other comorbidities*	30.5 (84)	30.3 (44)	30.8 (40)	.939
Any comorbidity	64.7 (178)	63.9 (92)	65.6 (86)	.760

Abbreviations: CVD, Cardiovascular disease; DM, Diabetes mellitus; HTN, Hypertension.

* Including: cancer, rheumatism, immunodeficiency or chronic respiratory, hepatic and blood diseases.

The most common complaints on admission, in order by frequency, were cough (48.4%), tiredness (41.0%) and fever (40.3%). Besides, O2 saturation less than “93%” were observed in 55.0% (*n* = 153) of patients. About 65% (178) of patients reported at least, one comorbidity the most common of which was hypertension (34.2%), followed by diabetes mellitus (31.5%) and cardiovascular disease (21.6%).

Out of 290 patients, 82 (28.3%) received ICU care, 43 (14.8%) required invasive mechanical ventilation, and 55 (19.0%) died during hospitalization.

Compared to patients without vitamin D deficiency, the proportion of males was significantly higher in those with vitamin D deficiency (63.4% versus. 50.0%, *p*‐value=.022); other assessed characteristics, including prevalence of underlying diseases, were similar between the two groups (all *p*‐values >.05).

Regarding the assessed poor outcomes of COVID‐19, higher percentage of patients with vitamin D deficiency died (27.7% versus. 14.3%; *p*‐value=.005), received ICU care (37.8% versus. 23.4%; *p*‐value=.006) and needed invasive mechanical ventilation (19.9% versus. 11.0%, *p*‐value=.036).

Table 2 shows the laboratory findings of patients at the first day of admission, overall and by vitamin D deficiency status. There was no statistically significant difference between the two groups regarding the assessed laboratory parameters.

**TABLE 2:** Laboratory findings of the study population on admission, overall and by vitamin D

Characteristics	Total	Without vitamin D deficiency	With vitamin D deficiency	*p*‐value
WBC count (×10^9^/L), Median (IQR)	6.1 (4.6–8.4)	5.7 (4.3–8.2)	6.7 (4.9–8.9)	.143
Lymph (×10^9^/L), Median (IQR)	1.9 (1.3–2.6)	2.0 (1.4–2.6)	1.8 (1.2–2.5)	.594
AST (U/L), Median (IQR)	38.0 (29.0–55.7)	37.0 (28.0– 53.5)	38.0 (29.0–63.0)	.996
ALT (U/L), Median (IQR)	28.0 (19.0–44.7)	30.0 (19.0–45.0)	28.0 (19.0–44.0)	.551
Creatinine (mg/dl), Median (IQR)	1.10 (0.9–1.3)	1.00 (0.9–1.3)	1.10 (0.9–1.3)	.584
LDH (U/L), Median (IQR)	590.5 (469.5–824.7)	563.5 (481.2–820.3)	614.0 (454.0–830.5)	.345
Hb (g/dl), Median (IQR)	13.1 (11.5–14.4)	13.2 (11.8–14.5)	12.8 (11.0–14.3)	.209
ESR (mm/h), Median (IQR)	41.0 (26.5–58.7)	40.0 (25.0–55.0)	42.5 (30.0–63.3)	.144
CRP >3+, % (*N*)	20.0% (50)	18.6% (24)	21.5% (26)	.569
CPK (U/L), Median (IQR)	163 (75–286)	170.5 (75.0–287.0)	145.0 (70.0–274.0)	.823
PT (S), Median (IQR)	13.5 (13.0–14.00)	13.5 (13.0–14.45)	13.5 (13.5–15.40)	.233

Abbreviations: ALT, alanine transaminases; AST, aspartate transaminases; CPK, creatine phosphokinase; CRP, C‐reactive protein; ESR, erythrocyte sedimentation rate; Hb, hemoglobin; IQR, interquartile range; LDH, Lactate dehydrogenase; Lymph, lymphocyte count; PT, prothrombin time; WBC, White blood cells.

Table 3 presents associations between vitamin D deficiencies with the poor outcomes of COVID‐19 separately, including death, need to ICU care and invasive mechanical ventilation based on the results of logistic regression models. Results from univariate logistic regression analysis showed that COVID‐19 patients with vitamin D deficiency were more likely to die (Crude OR [95% CI]: 2.30 [1.25–4.26]), require ICU care (2.06 [1.22–3.46]) and invasive mechanical ventilation (2.03 [1.04–3.93]) than those without vitamin D deficiency; however, in the multivariate logistic regression analysis, after considering potential confounders such as age and gender, the association of vitamin D deficiency with being intubated did not reach to the statistical significance (adjusted OR [95% CI]: 1.84 [0.93–3.62]; *p*‐value=.084).

**TABLE 3:** Area under the curve of Laboratory test for predicting death and requiring ICU care in patients with COVID‐19

Test	Death		Need to ICU care		Need to invasive mechanical ventilation	
	AUC (95% CI)	*p*‐value	AUC (95% CI)	*p*‐value	AUC (95% CI)	*p*‐value
WBC count, ×10^9^/L	0.59 (0.49–0.69)	.044	0.61 (0.53–0.69)	.004	0.57 (0.46–0.68)	.121
Lymph, ×10^9^/L	0.71 (0.62–0.79)	<.001	0.61(0.53–0.68)	.006	0.70 (0.60–0.76)	<.001
Hb, g/dl	0.63 (0.54–0.73)	.003	0.59 (0.51–0.67)	.016	0.60 (0.50–0.70)	.038
Creatinine, mg/dl	0.72 (0.64–0.81)	<.001	0.66 (0.58–0.73)	<.001	0.70 (0.61–0.80)	<.001
AST, U/L	0.59 (0.50–0.68)	.040	0.56 (0.49–0.64)	.093	0.56 (0.46–0.66)	.213
ALT, U/L	0.50 (0.41–0.59)	.991	0.46 (0.39–0.54)	.344	0.44 (0.35–0.53)	.229
LDH, U/L	0.64 (0.54–0.74)	.006	0.59 (0.50–0.68)	.033	0.60 (0.46–0.72)	.073
CPK, U/L	0.68 (0.58–0.78)	.001	0.57 (0.48–0.66)	.133	0.69 (0.58–0.79)	.001
ESR, mm/h	0.46 (0.37–0.56)	.420	0.48 (0.40–0.56)	.548	0.50 (0.40–0.60)	.968
PT, S	0.72 (0.64–0.80)	<.001	0.68 (0.61–0.75)	<.001	0.68 (0.58–0.77)	<.001
Vitamin D, ng/ml	0.63 (0.54–0.72)	.003	0.57 (0.50–0.65)	.051	0.61 (0.52–0.71)	.017

Abbreviations: ALT, alanine transaminases; AST, aspartate transaminases; CPK, creatine phosphokinase; ESR, erythrocyte sedimentation rate; Hb, hemoglobin; LDH, lactate dehydrogenase; Lymph, lymphocyte count; PT, prothrombin time; WBC, White blood cells.

**FIGURE 1:**
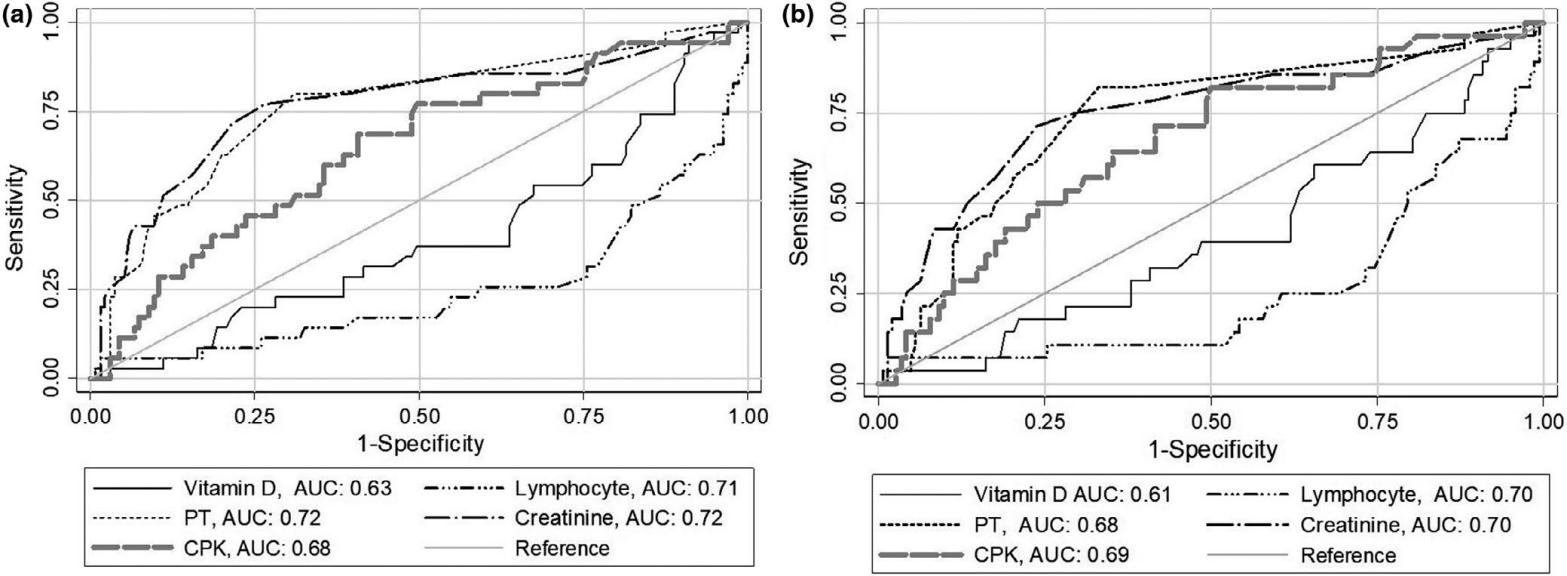
Receiver operating characteristic curves of vitamin D deficiency and blood parameters for predicting COVID‐19 death (a) and need to ICU care (b). CPK, Creatine phosphokinase; PT, prothrombin time

Table 4 shows AUC and its 95% confidence interval (CI) of laboratory parameters including vitamin D concentrations for predicting poor outcomes of COVID‐19. Vitamin D concentrations was among the parameters with the significant diagnostic accuracy for the early detection of COVID‐19 death (AUC (95% CI: 62.9 [54.1–71.7]) and need to ICU care (61.3 [51.7–71.0]); its diagnostic accuracy in detecting need to invasive mechanical ventilation was marginally nonsignificant. However, vitamin D concentration had significantly lower predictive ability compared to lymphocyte count, creatinine and CPK in the predicting COVID‐19 death and requiring ICU care (*p*‐values <.001, Figure 1).

**TABLE 4:** Prognostic impact of vitamin D deficiency for need to ICU, need to invasive mechanical ventilation and death following COVID‐19: Logistic Regression Analysis

Variable	Death		Need to ICU		Need to invasive mechanical ventilation	
	Crude OR (95% CI)	Adjusted* OR (95% CI)	Crude OR (95% CI)	Adjusted* OR (95% CI)	Crude OR (95% CI)	Adjusted*OR (95% CI)
Vitamin D deficiency	2.30 (1.25–4.26)^a^	2.0 (1.03–3.83)^a^	2.06 (1.22–3.46)^a^	1.83 (1.07–3.14)^a^	2.03 (1.04–3.93)^a^	1.84 (0.93–3.62)

Abbreviations: CI, Confidence Interval; OR, Odds Ratio.

^a^ *p*‐value <.05.

* Adjusted for age and sex.

## 4 DISCUSSION

According to our study, COVID‐19 patients with vitamin D deficiency (a serum level of less than 20 ng/dl) were about twice as much at risk for ICU hospitalization and death, even after adjusting the statistical model for age and gender.

In a recent study, linking the data from UK Biobank to COVID‐19 test results revealed an association between vitamin D and COVID‐19 infection which was not significant after adjustment for confounders. Among the 348,598 participants, 449 tested positive for COVID‐19. The median serum 25(OH)D concentration was lower in COVID‐19 positive patients. However, after performing univariable logistic regression analysis adjusted for confounding factors, the authors found no strong evidence to support a potential protective role of vitamin D against COVID‐19 infection (Hastie et al., 2020).

Another retrospective multicentral study of 212 cases with laboratory‐confirmed infection of SARS‐CoV‐2 showed that serum 25(OH)D level was lowest in critical cases, and a decrease in serum 25(OH)D level could worsen clinical outcomes of the patients (Alipio, 2020).

Meltzer et al. studied the documents of 4314 patients tested for COVID‐19. Of those, 499 had had a vitamin D result during the last year, and 178 (36%) cases were diagnosed as vitamin D deficient. The multivariable analysis suggested that persons with vitamin D deficiency were at a substantially higher risk of testing positive for COVID‐19 (Meltzer et al., 2020).

Several possible mechanisms can link vitamin D to COVID‐19. Firstly, it is now clear that vitamin D plays important roles in the modulation of the immune system and its low level is associated with both increased autoimmune and infectious diseases. Calcitriol, the active form of vitamin D, has its own nuclear receptor (VDR) through which can alter gene expression. To our knowledge, immunologic cells are capable of synthesizing the active calcitriol, and the VDRs have been found in these cells as well. Therefore, vitamin D can even act in a paracrine or autocrine manner to affect immunity (Aranow, 2011).

Vitamin D improves physical protection against pathogens by maintaining junction integrity (Schwalfenberg, 2011). It also enhances cellular innate immunity partly through the induction of antimicrobial peptides (such as cathelicidin and defensins) as well as reducing the cytokine storm which has been widely observed in COVID‐19 patients (Huang et al., 2020). Vitamin D is a modulator of adaptive immunity via suppressing responses mediated by the T helper cells type1 (Th1), promoting cytokine production by the T helper cells type 2 (Th2) and improving induction of the T regulatory cells, thereby inhibiting inflammatory processes (Cantorna et al., 2015). It therefore comes as no surprise why many documents depict an association between lower vitamin D levels and a higher rate of upper respiratory tract infection, influenza, bacterial vaginosis, HIV and other viral or microbial infections (Cannell et al., 2006; Ginde et al., 2009; Laaksi et al., 2007; Rodriguez et al., 2009; Villamor, 2006).

A recent study claimed that subtropical and mid‐latitude countries are most affected by COVID‐19. This finding, if correct, might be partly explained by the higher prevalence of vitamin D deficiency in these geographical regions (Rhodes et al., 2020). Furthermore, the seasonal fluctuation of serum vitamin D level might be responsible for the higher risk of respiratory infections in fall and winter (Juzeniene et al., 2010).

Another link between COVID‐19 and vitamin D is the rennin angiotensin system (RAS). It seems that angiotensin converting enzyme 2 (ACE2) is the functional receptor of SARS‐CoV‐2 to which the virus gets attached and then penetrates into the host cell. During the SARS‐CoV‐2 infection, tissues, especially lungs, face a loss in ACE2 function followed by an increase in the concentration of angiotensin II which finally leads to increasing alveolar permeability and accelerating lung damage (Rashedi et al., 2020). Interestingly, vitamin D can increase the expression of ACE2 mRNA which can act as a double‐edged sword; on the one hand, it can increase the number of receptors for the virus to enter the cells and on the other hand it can protect the pulmonary tissue from damage by saving the function of ACE2.

Low vitamin D concentration was among the parameters with the significant diagnostic accuracy for the early detection of COVID‐19 death and requiring invasive mechanical ventilation. Vitamin D seems to have the same or more predictive value than LDH, ESR and CRP for COVID‐19 poor outcomes. However, it has lower predictive ability compared to lymphocyte count, creatinine and CPK. Previously, independent parameters such as underlying comorbidity, older age, higher LDH and lower lymphocyte count were used to develop a scoring model for predicting the progression of COVID‐19 (Ji et al., 2020).

In conclusion, the present study showed that vitamin D deficiency can be considered as a predictor for poor outcomes and mortality in COVID‐19 patients. Therefore, taking vitamin D supplements according to the prophylactic or treatment protocols is recommended as before. In addition, it is suggested that serum vitamin D status be checked in all COVID‐19 patients on admission and appropriate action be taken to correct the possible deficiency or insufficiency.

## CONFLICT OF INTEREST

All contributing authors declare that they have no conflicts of interest.

## ETHICAL APPROVAL AND CONSENT TO PARTICIPATE

Ethics approval for the study protocol was granted by The Human Ethics Committee of Alborz University of Medical Sciences (ABZUMS) and Iran University of Medical Sciences (IUMS). All participants signed written informed consent forms.

## CONSENT FOR PUBLICATION

The authors would like to advise that all authors listed have contributed to the work. All authors have agreed to submit the manuscript to Food Science and Nutrition. No part of the work has been published before.

## Data Availability

The data are not publicly available because of containing information that could compromise the privacy of research.
